# A pilot study to estimate the population size of endangered Galápagos marine iguanas using drones

**DOI:** 10.1186/s12983-022-00478-5

**Published:** 2023-01-26

**Authors:** Andrea Varela-Jaramillo, Gonzalo Rivas-Torres, Juan M. Guayasamin, Sebastian Steinfartz, Amy MacLeod

**Affiliations:** 1 Institute of Biology, Molecular Evolution and Systematics of Animals, University of Leipzig, Leipzig, Saxony Germany; 2 Laboratorio de Biología Evolutiva, Colegio de Ciencias Biológicas y Ambientales COCIBA, Instituto Biósfera, Universidad San Francisco de Quito USFQ, Calle Diego de Robles s/n y Pampite, Cumbayá, Pichincha, Quito Ecuador; 3 Galápagos Science Center, GSC, San Cristóbal, Galápagos, Ecuador; 4 Wildlife Ecology and Conservation, University of Florida, FL Gainesville, USA; 5 3Diversity, Quito, Pichincha, Ecuador

**Keywords:** Aerial photography, Conservation, Drones, Monitoring, Population survey, Survey methods, Technology, UAVs

## Abstract

**Background:**

Large-scale species monitoring remains a significant conservation challenge. Given the ongoing biodiversity crisis, the need for reliable and efficient methods has never been greater. Drone-based techniques have much to offer in this regard: they allow access to otherwise unreachable areas and enable the rapid collection of non-invasive field data. Herein, we describe the development of a drone-based method for the estimation of population size in Galápagos marine iguanas, *Amblyrhynchus cristatus*. As a large-bodied lizard that occurs in open coastal terrain, this endemic species is an ideal candidate for drone surveys. Almost all *Amblyrhynchus* subspecies are Endangered or Critically Endangered according to the IUCN yet since several colonies are inaccessible by foot, ground- based methods are unable to address the critical need for better census data. In order to establish a drone-based approach to estimate population size of marine iguanas, we surveyed in January 2021 four colonies on three focal islands (San Cristobal, Santa Fe and Espanola) using three techniques: simple counts (the standard method currently used by conservation managers), capture mark-resight (CMR), and drone-based counts. The surveys were performed within a 4-day window under similar ambient conditions. We then compared the approaches in terms of feasibility, outcome and effort.

**Results:**

The highest population-size estimates were obtained using CMR, and drone-based counts were on average 14% closer to CMR estimates—and 17–35% higher—than those obtained by simple counts. In terms of field-time, drone-surveys can be faster than simple counts, but image analyses were highly time consuming.

**Conclusion:**

Though CMR likely produces superior estimates, it cannot be performed in most cases due to lack of access and knowledge regarding colonies. Drone-based surveys outperformed ground-based simple counts in terms of outcome and this approach is therefore suitable for use across the range of the species. Moreover, the aerial approach is currently the only credible solution for accessing and surveying marine iguanas at highly remote colonies. The application of citizen science and other aids such as machine learning will alleviate the issue regarding time needed to analyze the images.

## Introduction

Quantification of individuals in wild populations is of foremost importance for ecologists and conservationists; given the rapid and ongoing biodiversity crisis, this need has never been greater [1]. For species of conservation concern, a lack of frequent and accurate population-size surveys can preclude an understanding of the trajectories of populations, thus greatly hampering effective management [2]. However, gaining accurate population-size estimates for many species is a significant challenge, and performing classical ground-based techniques often requires more time, funds, and access than is available—this is especially true for species occurring in remote regions, or where capacity for such work is severely limited [3, 4]. As an alternative to ground-based methods, aerial surveys have become a standard technique in wildlife monitoring. Unmanned Aerial Vehicles (UAVs or drones) are increasingly used for this purpose, since they are relatively cheap to use and allow the collection of high-quality images, which can be later analyzed in a multitude of ways [5–7]. Drones are now readily available, typically do not require high levels of specialist training to pilot, and give visual access to remote locations that are impossible to reach by other means. Several examples proving the efficacy of drone-based monitoring in a variety of habitats now exist, covering a range of terrestrial and marine species, such as crocodiles [8], sea turtles [9], lizards [10], sharks [11], and birds [12, 13]. Crucially, drones offer a means by which to reach threatened and understudied species in inaccessible terrains, such as those living in remote island localities. Though drone surveys are relatively non-invasive, there is the possibility that they create stress in wild animals. However, recent studies have shown this not to be the case [14], but this depends on the species and proximity of the drone [15].

The Galápagos marine iguana (*Amblyrhynchus cristatus*) is an endemic and emblematic inhabitant of the world-renowned Galápagos Archipelago. In recognition of its limited distribution range and mounting anthropogenic threats, the marine iguana is listed as Vulnerable to extinction on the IUCN Red List of Threatened Species, with six of the 11 subspecies considered Endangered and four recognized as Critically Endangered [16]. Ongoing anthropogenic threats include predation by introduced invasive species like feral cats (*Felis catus*), pigs (*Sus scrofa*), dogs (*Canis familiaris*), and black rats (*Rattus rattus*) [17–19], as well as marine pollution [20]. Potentially important emergent threats include increasingly severe El Niño-linked starvation events resulting from climate change [21, 22], and increasing levels of tourism and urbanization [23]. Despite its status as a world heritage site and the strenuous efforts to protect its unique biodiversity, the Galápagos Archipelago is changing rapidly, and exponential levels of growth are forecast for numbers of both tourists and permanent residents [24]. The ongoing rise in pollution and alien invasive species are a direct result of human population growth, which threatens the survival of some endemic species [25].

**Fig. 1:**
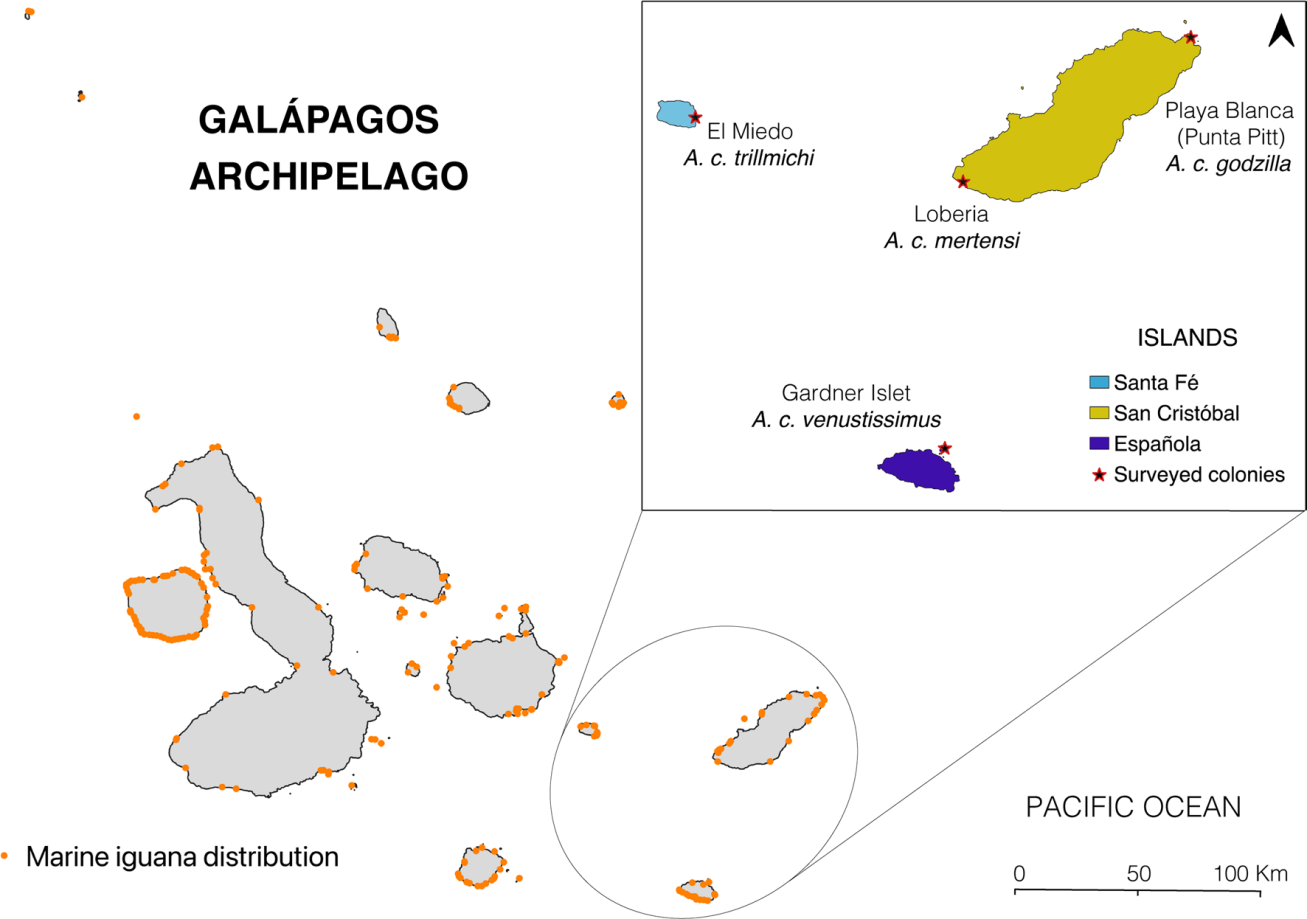
Map of the four colonies surveyed for marine iguana population size on the Galápagos Archipelago (Ecuador) in January 2021; corresponding subspecies are given for each site. Marine iguana distribution data taken from Arteaga & Guayasamin [19]

The paramount challenge for marine iguana conservation lies within the scarcity of data on the location, size, and health of colonies. For most subspecies, the only available estimates of population sizes are from 2004; numbers which are stated to be “very rough” by the authors of that work [26]. The Galápagos National Park authority (GNP) undertakes regular wildlife surveys where some marine iguana colonies are monitored, and their health assessed. In their annual report from 2019 they register 25,691 individuals as censused on 17 colonies across the entire archipelago, and in December 2021 they report 27,758 individuals visually counted from 33 colonies on the 13 main islands [27, 28]. Although this information is certainly useful, these censuses cover only selected accessible localities, and the methods used may also only capture a proportion of individuals at any given site [29], therefore they only represent a sample of the whole population. Complete and reproducible estimates are urgently needed for long-term monitoring and assessment of conservation status at both the species and subspecies level. The grounds for the lack of data on population sizes is simple—since the marine iguana distribution range spans the entire archipelago (Fig. 1), it poses a special logistical challenge to surveyors. A full population survey by traditional means would be an extremely expensive and time-consuming endeavor, with potential risks for both the surveyors and the islands. Many marine iguana colonies are difficult—if not impossible—to reach, due to a lack of safe access for boats (e.g., colonies situated on cliffs), and surveys by foot are both slow and potentially dangerous because of the extremely sharp and unstable lava-rock underfoot. These issues, coupled with challenging sea conditions, increase the complexity of the endeavor. Furthermore, any surveys by foot—no matter how carefully done—run the risk of introducing non-native and potentially invasive organisms to these sensitive pristine sites [25]. Consequently, a complete and scientifically rigorous archipelago-wide population survey of this species by classical approaches is not feasible. We therefore undertook to develop a new approach—using Unmanned Aerial Vehicles (UAVs), hereafter referred to as drones—that is capable of estimating population sizes of marine iguanas across their complete range for the first time; the pilot stage of this work is described herein. Given their large body size, sedentary nature, and remote coastal habitat, marine iguanas are prime candidates for aerial surveys, and the population size estimates gained will be invaluable for directing effective efforts to protect this iconic species.

We collected aerial photographs of different colonies representing four distinct taxonomic units (subspecies) of marine iguanas from three main islands in the Galápagos. We then counted the iguanas from orthophotographs and compared the results to the outcomes of two traditional ground-based methods—capture mark-resight (CMR) and simple counts, which were performed in parallel. In doing so, we test the feasibility of the field methods and investigate whether the drone-based approach can produce images from which marine iguanas can be reliably identified. Our overall aim was to assess the potential of this new method for solving the logistical problems inherent in archipelago-wide surveys of the Galápagos marine iguana, and in a wider sense, provide a case study for the use of drones—flown from boats—for surveying species in remote and inaccessible coastal terrains. We expect that drone-based surveys will take less time in the field and allow access to more locations than traditional methods but will require more time for post-processing.

## Results

### Drones, image collection and processing

We were able to fly the drones reliably and safely at all localities in varying sea and weather conditions, without significant wildlife conflicts. We therefore confirm that it is entirely feasible to perform aerial surveys from a boat for the purpose of monitoring marine iguanas in the Galápagos, even despite the limited previous experience of the drone pilot in this study. We completed 34 flights, covering a total of 6.7 km of coastline and 11:07 flight hours, flying at a height of 20-25 m. Although higher altitudes increase survey speed, the iguanas captured on the images can become too small to reliably identify, making counting errors more likely. The best flight altitude is, therefore, a trade-off between survey efficiency and detection probability. We found that, with proper training, a non-expert pilot can safely fly the drones and manually achieve the same data quality as those obtained from automated flights (i.e., those controlled automatically by software). Moreover, manual flights were better for monitoring this type of habitat because the pilot has finer control over the flight path and can more effectively select the target survey area, e.g., to adjust to changing tides. We preferentially launched drones from the boat in most locations, though occasionally chose to survey from beaches accessible by foot from the town, or when sea and wind conditions were difficult and safe boat landings were readily available (e.g., Loberia in San Cristobal and El Miedo on Santa Fé). Surveying from the boat was faster than from land because we were able to easily move and follow the drone along the coastline by boat, whereas land-based launches require the pilot to walk towards the next launching point. For example, for the same distance, it took 5.20 h to survey La Lobería (drones launched from land), while for Gardner islet (surveyed by boat), we needed 3.50 h. The advantages of flying directly from the boat were most evident when the terrain to be covered included sharp and structurally complex lava rock. We also observed that the iguanas were less disturbed by the presence of the drones than by humans (pers. obs. AVJ, AM), and this was evident regardless of whether the site was touristic or not; presumably therefore drone-use reduces the probability of missing some iguanas—who typically run and hide within lava cracks in response to nearby humans—when compared with foot surveys. It is inherently difficult to quantify and compare the disturbance caused by foot-based vs. drone-based surveys because it is likely that animals were hiding before being seen, and thus ‘hiders’ could therefore not be counted. However, we observed that far fewer individuals reacted visibly to the drones (by looking up and moving, but generally not hiding) than to our physical presence on the ground. Very few bird encounters are worth remarking on (though see discussion). Additionally, we registered a variety of plant and animal species—as well as plastic objects and other refuse—on the images.

**Fig. 2:**
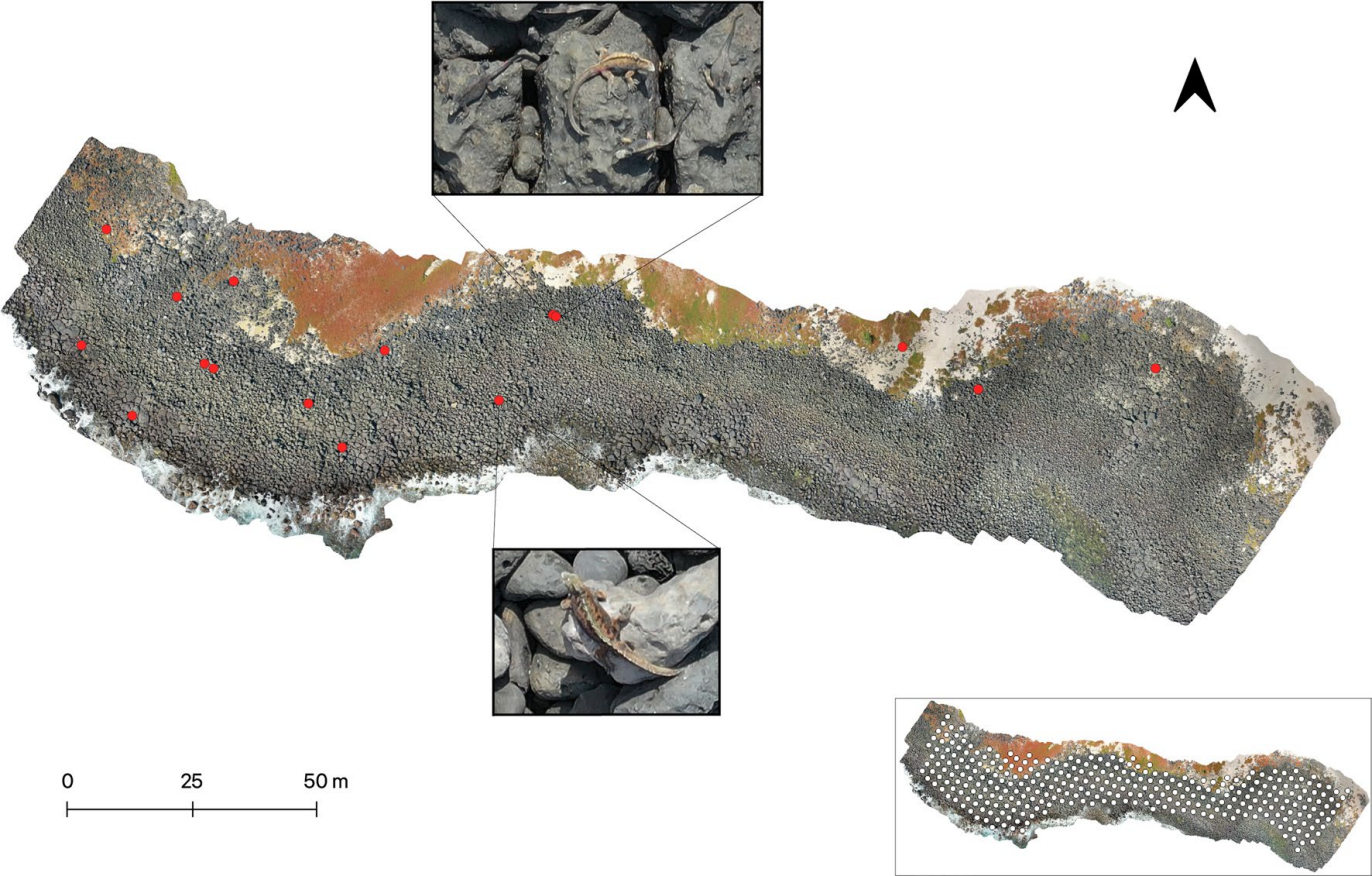
Example of an orthophotograph (2D map reconstruction) from La Lobería, San Cristóbal Island, Galápagos, Ecuador. The red circles represent individual marine iguanas identified, as is shown in the enlarged boxes. The above box shows a lek (reproductive group) and the box below shows a solitary reproductive adult male. Bottom right box shows the flight path of the drone, with points denoting locations where photographs were taken

Orthophotographs provided a sound basis from which to count and geographically locate marine iguanas (Fig. 2). Having the iguanas marked in the orthophotograph also gives the option to verify and compare data from multiple observers. We found that when the images have sufficient overlap (low overlap can create holes or blurred areas in the reconstruction), the observable resolution of the orthophotograph was satisfactory regardless of whether it was created under low-, medium- or high-quality parameters. By using a medium-range computer (12GB RAM memory and an Intel Core i7), low quality settings required on average 2–3 h. for the complete process, medium quality 8–9 h. and high quality up to 3 days. Based on the similarity of results, we recommend using low or medium quality settings to save time and create smaller files to work with later.

### Aerial- versus ground-based (classical) counts

Regarding time spent in the field, the ANOVA test yielded variation among methods (df = 2, *P *< 0.01). On average, collection of images for aerial surveys took less time (mean 2.6 ± 1.1 h. per colony) than did simple counts (mean 3.4 ± 1.2 h.), though this difference was not significant (post hoc Tuckey test; *P * = [0.75], 95% C.I. = [− 2.16, 3.72]). However, clear differentiation is observable in El Miedo (Santa Fe), a site with difficult terrain, where we needed one hour with the drones versus 3 h for simple counts to complete the ~ 650 m of coastline covering the colony. Similar results were found for Gardner islet (Española)—where the ground-based method took almost double the time (See Table 1). Time needed to survey by drone was significantly lower than that required to perform the CMR (mean 7.4 ± 1.9 h.) (post hoc Tuckey test= (*P * < [0.01], 95% C.I. = [− 7.75, − 1.88]). This result is expected, as CMR is a two-occasion method that required the animals to be captured and marked. Similar results were observed in comparisons between simple counts and CMR (post hoc Tuckey test= (*P * = [0.01], 95% C.I. = [− 6.97, − 1.87]). Post-processing the aerial images took an average of 22 h. (± 9.1) per site and counting the iguanas from the orthophotographs required on average 26.3 h. (± 10.3). Though this was considerably longer than the time needed for counting on the ground (on average 7.3 h ± 1.5), it should be noted that post-processing time is mostly computational and does not require much user input or effort.

**Table 1:** Comparison of the survey time required in the field for each method used, and number of marine iguanas estimated/counted at four colonies in the Galápagos Archipelago, Ecuador

Locality	Survey time in the field (h.)			Marine Iguanas counted		CMR	
	CMR	Simple counts (ground)	Aerial counts (drones)	Simple counts (ground)	Aerial counts (drones)	N (CI)	SE
La Lobería (San Cristóbal Island)	9:30	3:30	3:34	102	138	161 (145–177)	8.37
Playa Blanca (San Cristóbal Island)	5:36	2:21	3:37	60	70	78 (69–87)	4.45
El Miedo (Santa Fe Island)	6:20	3:10	1:00	252	285	468 (426–510)	21.50
Gardner Islet (near Española Island)	8:57	5:13	2:56	215	260	373 (339–407)	17.07

Results are given for three methods: aerial counts using drones, simple counts performed on the ground, and from Capture-Mark-Resight (CMR), where N represents the population size estimate, SE is the standard error and CI are the confidence intervals for N

**Fig. 3:**
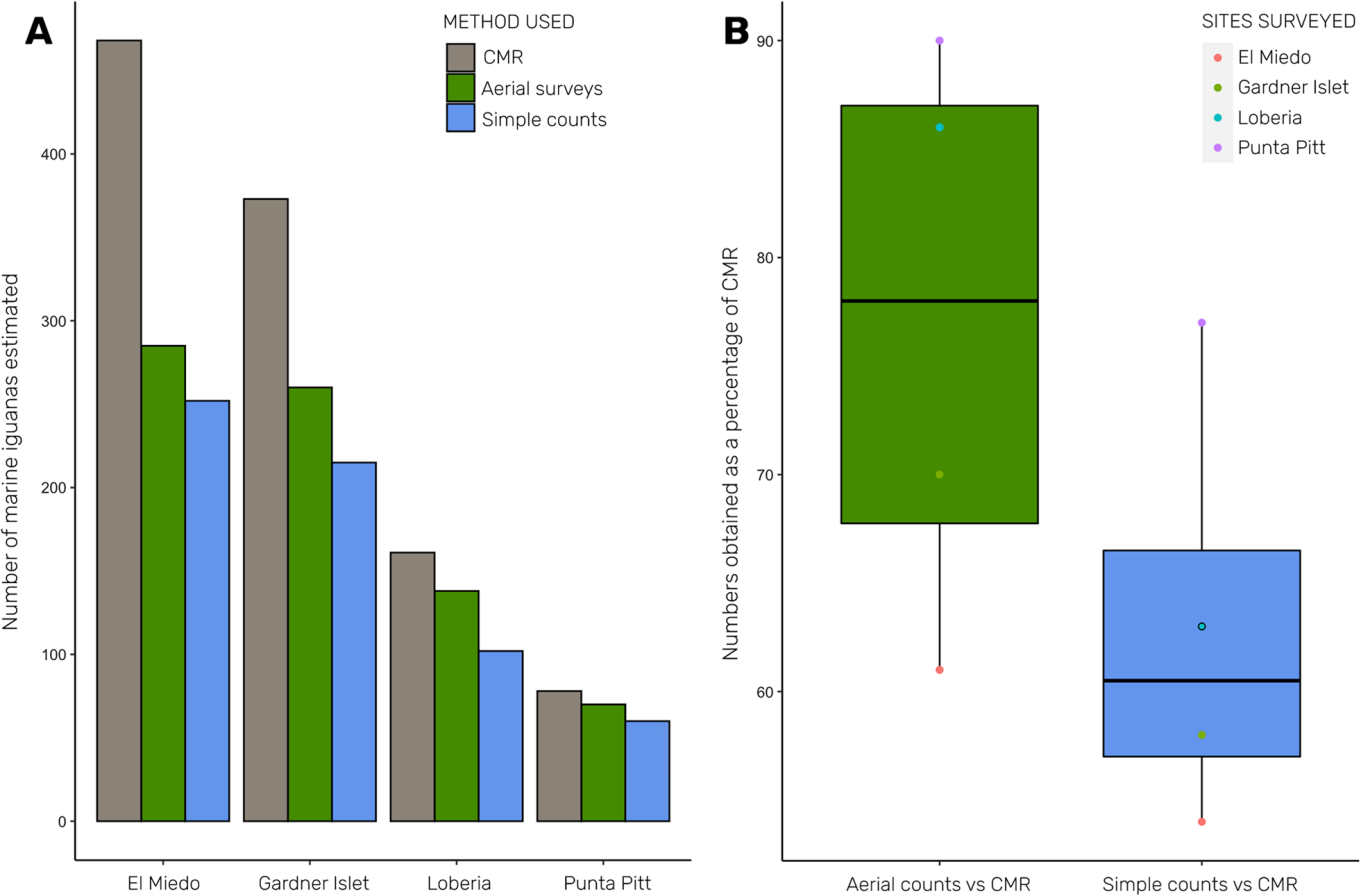
Graphic showing comparisons of methods used against marine iguana abundance. **A** Bar plot comparing the marine iguana abundance (number of individuals estimated/counted) among the three sampling methods used for all sites sampled; **B** Box plot comparing the numbers of marine iguanas counted (as percentages) during surveys using aerial and simple counts approaches as compared to estimates gained using the capture mark-resight (CMR) method

We found that marine iguanas were visible enough on the photos to enable accurate counts, especially when the observer has some experience (Fig. 2). The number of marine iguanas counted from aerial surveys (av. 188 ± 101.7) was higher than the number from simple counts (av. 157 ± 90.9) for all the sites surveyed. Although these differences were not statistically significant (Chi^2^ test, *P* = 0.72). Notably, both drone-based and simple count estimates are lower than those obtained with CMR (Table 1; Fig. 3a). This was not surprising as the CMR method corrects for non-detected individuals (e.g., those sheltering under rocks or out at sea). However, results from the drone method are 17–35% higher than those obtained from simple counts and were therefore closer—by 14% on average—to the CMR estimates at all colonies. At higher densities, the drone and simple count methods appear to more seriously underestimate the population size when compared to CMR results. For instance, the aerial counts captured 90% of the CMR estimates at the low-density colony of Playa Blanca, but only 60% at El Miedo where iguana abundance is high. This issue was even more pronounced in the ground-based simple count approach, which captured 77% of the CMR estimate at Playa Blanca, and just 54% at El Miedo (Fig. 3b).

## Discussion

### Drone based approach shows great promise for population surveying

The advantages of drones over traditional methods have already been acknowledged [2, 30, 31], and our work further supports the notion that drone surveillance is well-suited for the task of monitoring endangered biodiversity in remote and protected areas. Several recent studies have highlighted drones as an innovative and valuable approach for conservation research in other Galápagos species [32–34], and we herein confirm the feasibility of this method for conducting non-invasive aerial surveys of marine iguanas. This species is ideal for drone monitoring because of their basking behavior—where they gather in exposed locations on the coast—and are highly visible from the air. Also, they accumulate in relatively narrow sections of the coastline, thus geographically limiting the survey area. Furthermore, tails from marine iguanas resting or hiding from the sun under the rocks (a common behavior) are most visible from directly above, and such individuals are therefore more detectable by drones than by a person walking through colonies. Furthermore, we observed that the iguanas were less disturbed by drones than by people, resulting in fewer individuals hiding during aerial surveys; this is in keeping with research in another remote localities [35]. This effect was especially true for smaller individuals, and in areas where predation by invasive species is common and iguanas tend to be more skittish [18]. An additional important consideration is that drones help to avoid “convenience sampling” [36] because they greatly facilitate access to remote sites and thereby allow surveys of the whole known potential distribution of a species, with a high probability of finding previously unreported colonies. Indeed, our own experiences confirm this to be the case on Santa Fé island, where several new sites of occurrence were recorded.

In a wider sense, we found that our images showed other cohabiting species, such as sea lions (*Zalophus wollebaeki*), crabs (*Grapsus grapsus*), some species of birds (e.g. *Pelecanus occidentalis urinator, Sula nebouxii, S. sula, S. granti, Creagrus furcatus*) and fish (e.g. *Rhinoptera steindachneri, Aetobatus narinari, Prionurus laticlavius*), green turtles (*Chelonia mydas*), coastal plants (*Opuntia galapageia, Scalesia* spp.) and algae (e.g. *Gelidium* spp., *Phaeophyta* spp.). Our dataset could therefore be of use in studies of other species, both now and in the near future. We also found high concentrations of plastic pollution over certain parts of the coast; these images could facilitate further research on aggregation patterns [37] and inform possible refuse collection campaigns.

### Pros and cons of the drone method

On average, drone surveys were completed faster than simple counts in the field, though the differences were not statistically significant. However, it is important to mention that we did not consider the time required in the field for finding a suitable boat landing site, disembarkation, and time spent walking to the survey sites—all time-consuming aspects of traditional field surveys that can be avoided when using drones. Moreover, we could not quantify surveyor risk when landing on the islands and walking on these hazardous substrates, but our own experiences and discussions with locals tell us these hazards are very significant. In addition, it is worth noting again that this is a pilot study and thus by its very nature, included experimentation with methods and use of a new approach; this entailed significant extra time and work and thus it stands to reason that future drone surveys would be more efficient and thus quicker.

Although the statistical comparison of drone-based and ground-based simple counts was not significant, this may well be due to the small number of comparisons herein and the highly variable nature of the colony sizes; clearly further comparisons are needed. However, aerial surveys did detect more iguanas than traditional simple counts in all sites. Moreover, aerial surveys additionally offer potential for assessing and improving the accuracy of results, since single observer counts from images can be repeated, which is generally not the case when counting in the field. This should be especially useful in high density colonies, where surveyors will typically visually estimate the number instead of counting each animal (pers. comm. GNP managers). Beyond that, drone images have great potential for post-field research applications; since researchers can perform later analysis in a safer and more comfortable environment [38], thereby facilitating data uses that would not be possible in the field. For instance, in the images, we were able to identify reproductive adult males by noting their bright coloration and could distinguish breeding pairs, leks, and nearby lone reproductive males. This easily-collected information could allow us to study the reproductive behavior of the marine iguana without the need for further extensive fieldwork. Previous studies have already proven the potential of aerial images to not only monitor communities, but also to study their mating dynamics [39, 40], and with the proper ground calibration, the body sizes of individuals can be accurately measured [8], enabling us to distinguish age classes, sex, and potentially health status. Likewise, by georeferencing marine iguanas, we can study patterns of occurrence and aggregation across the islands and characterize their habitat quality [41]. This information can be used to highlight areas as critical sites for protection, further aiding the recognition of specific conservation priority areas in the Galápagos Archipelago [42].

With further development to reduce the effort needed for post-processing and counting, drone-based surveys should prove less expensive than traditional ones, enabling more frequent monitoring, which is urgently needed. For instance, comparing the outcome of our CMR estimates with those of MacLeod et al. [29], we see a reduction in population size by over half in two key colonies (La Lobería: N = 400/161 and Playa Blanca N = 183/78 in 2013/2021, respectively). As these studies were performed at different times of the year, we cannot conclude a population decline because the difference may only reflect interannual migrations/movement dynamics—something we know almost nothing about in marine iguanas. In any case, both sets of CMR results confirm the worryingly small size of the colonies studied—each of which constitutes the single largest colony of the two subspecies found on San Cristóbal Island (*A. c. mertensi* and *A. c. godzilla*).

Whilst we have shown that aerial surveys can be used to monitor marine iguanas, they do still underestimate population size when compared to the estimates provided by CMR. Naturally, CMR would be the method of choice if accuracy were the only consideration, but the field time and access required for CMR makes it completely unfeasible in the vast majority of marine iguana colonies. Further, in colonies of higher densities—like on Fernandina Island, where congregations of many hundreds of individuals are found— it would likely be impossible to mark enough individuals to gain good estimates using CMR. Moreover, the colonies surveyed would need to be well-studied in advance in order to avoid invalidating the assumptions of the CMR method, this is—at best—unrealistic for most populations. The most pragmatic approach could therefore be to establish whether the underestimation of animals using simple counts and drone counts is stable when compared to the more accurate outcome of CMR at certain focal sites. If so, we could then apply a correction factor to the underestimates which will allow us to obtain new estimates via aerial methods to compare with the historic records from simple counts that exist for some sites. Considering the type of terrain and densities of iguanas will be important here, as colony density and complexity of terrain appear to influence the reliability of results. To evaluate this further, we plan to repeat the comparisons described herein at more colonies across the archipelago. However, even if such comparisons cannot be performed, with the aerial method we can at least ensure that the more accurate data necessary for the estimation of population trajectories can be collected in the future, and that colonies which are inaccessible by foot can nonetheless still be surveyed.

Finally, the aerial images collected can serve as an archive of the current conditions, which will enable their future use for historical comparisons, and for new research questions which may arise. For instance, a recent project surveying *Opuntia* cacti on the Galápagos used historic photographs to compare against the contemporary situation and thereby measure the scale of *Opuntia* loss [43]. As our aerial photographs recorded not only marine iguanas but also many other species, man-made objects, and various aspects of the environment, they could prove invaluable for such uses in the future.

There are naturally also disadvantages to using drones. Though the drones did greatly facilitate fieldwork, our ability to fly them depended on daily conditions; both weather and sea conditions can be limiting factors [44], though naturally the same can be true for ground-based surveys. Fortunately, the season selected coincides with more stable weather conditions and calm seas, therefore we were almost always able to fly the drones. Furthermore, rapidly developing technology means that in the future, such limitations will be decreasingly important—indeed more expensive commercial drones can already fly in far more extreme conditions than we encountered in the field. A further challenge is due to the drones being relatively power-hungry and the rather short flight times as dictated by the battery capacity of consumer-level drones. This can be circumvented somewhat by simply bringing more batteries, but this may prevent their use when operating in very remote locations where electrical supply for charging is limited [45]; again, new technologies such as better batteries and solar chargers may alleviate this issue. Furthermore, using video rather than photography may use less power and allow the capture of other information, such as behaviors; we will investigate the use of video in future work. In terms of safety risks, we were initially concerned regarding potential disturbance and related conflicts with wildlife—such as breeding birds like the magnificent frigate bird (*Fregata magnificens*), which is common in our study area. Our experiences confirm earlier findings that it is solitary birds in flight—rather than those in large breeding colonies— that react to the drone presence [46]. Though individual birds occasionally approached the drones mid-air—potentially posing a threat both to themselves (from the propellers) and to the drones—we found this to be a small risk that could be managed by careful piloting and continually watching the drones during flights. We found that if the drone was approached, raising the flight altitude usually solved the problem (pers. obs. GRT, AVJ).

Overall, a key challenge was the relatively time-consuming tasks of managing and processing the large quantity of data collected, especially with regards to the manual counting of iguanas. In terms of saving time during analysis, we found that using a powerful computer is critical (recommended: Windows PC with 32–64 RAM memory, 4–8 core processors, and 8 VRAM graphic processing unit), for instance a process that took up to 102 h. could be reduced by a third or more when several orthomosaics can be built simultaneously. Manual counting is a highly laborious activity and could therefore represent a significant obstacle to the use of image-based data for monitoring practices. To this end, similar projects have proposed the use of artificial intelligence (AI) and crowdsourcing counting via Citizen Scientists [47–50]—use of these approaches could reduce the need for computational power and minimize the specialist workload; we intend to use both approaches in our forthcoming analysis and have already run a successful citizen science project via the Zooniverse platform (www.zooniverse.org). AI can be developed and trained to handle much of the post-processing—including the building of orthophotographs—and once fully trained, the algorithms should be capable of detecting biologically important objects like iguanas in future datasets with minimal need for additional human-input. Use of thermal imaging may also facilitate the visualization of the iguanas.

## Conclusions and outlook

Despite some challenges, we find that drones are highly applicable for the purpose of monitoring Galápagos marine iguanas, and that this novel approach boasts many advantages when compared to traditional methods, both for researchers and wildlife. The various rapidly developing technologies related to drone-use hold great promise for collecting realistic population-size estimates in a short time, and thus can greatly facilitate the conservation management of this species. Although this paper constitutes a case study specifically focused on the marine iguana, we believe that drone-based aerial surveys will prove useful for the study of other larger bodied organisms which occur in open habitats.

Our next steps include testing the use of drones across the archipelago in a wider variety of terrains and habitats (e.g., high cliffs, nesting areas) as these may present new challenges [51]. Furthermore, we seek to find the most efficient way of collecting and processing images, facilitating the use of this method for monitoring all 11 marine iguana subspecies across their entire distribution range; this will involve crowdsourcing data analysis via Citizen Science projects and the use of AI. Combining the two approaches may be ideal, as the inputs of human volunteers can be used to train the computers [6, 52], resulting in faster, more accurate results requiring less “expert” input and thereby freeing scientists and conservation managers from performing these time-consuming repetitive tasks and increasing the possibility that such surveys can be performed on a regular basis.

## Methods

### Area and subspecies surveyed

**Fig. 4:**
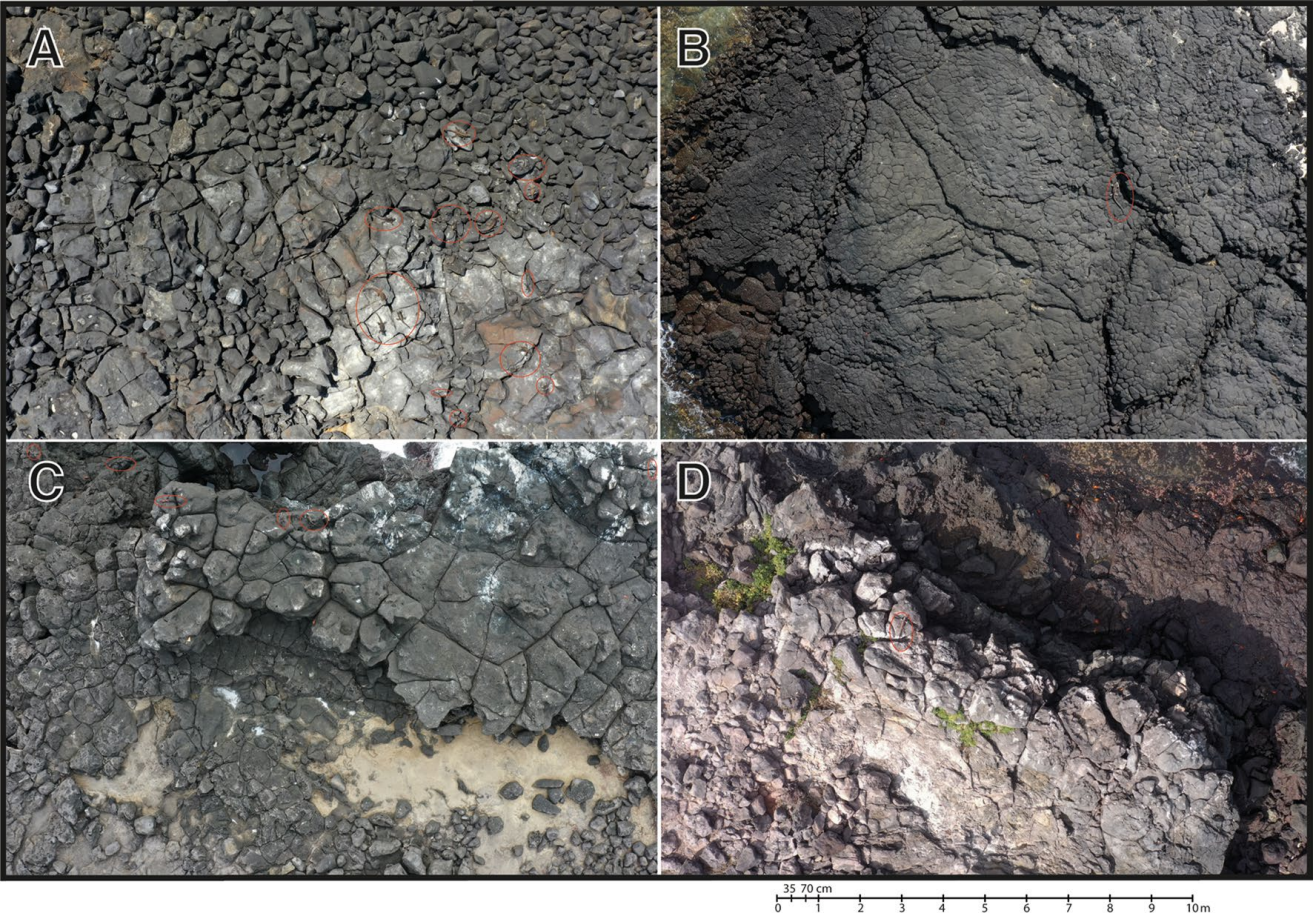
Characteristic images of the terrain for each colony surveyed for marine iguanas on the Galápagos islands (Ecuador) in January 2021. **A** La Lobería and **B** Playa Blanca (Punta Pitt): San Cristóbal Island; **C** El Miedo: Santa Fé Island, and **D** Gardner Islet: Española Island. Photographs: Andrea Varela

**Fig. 5:**
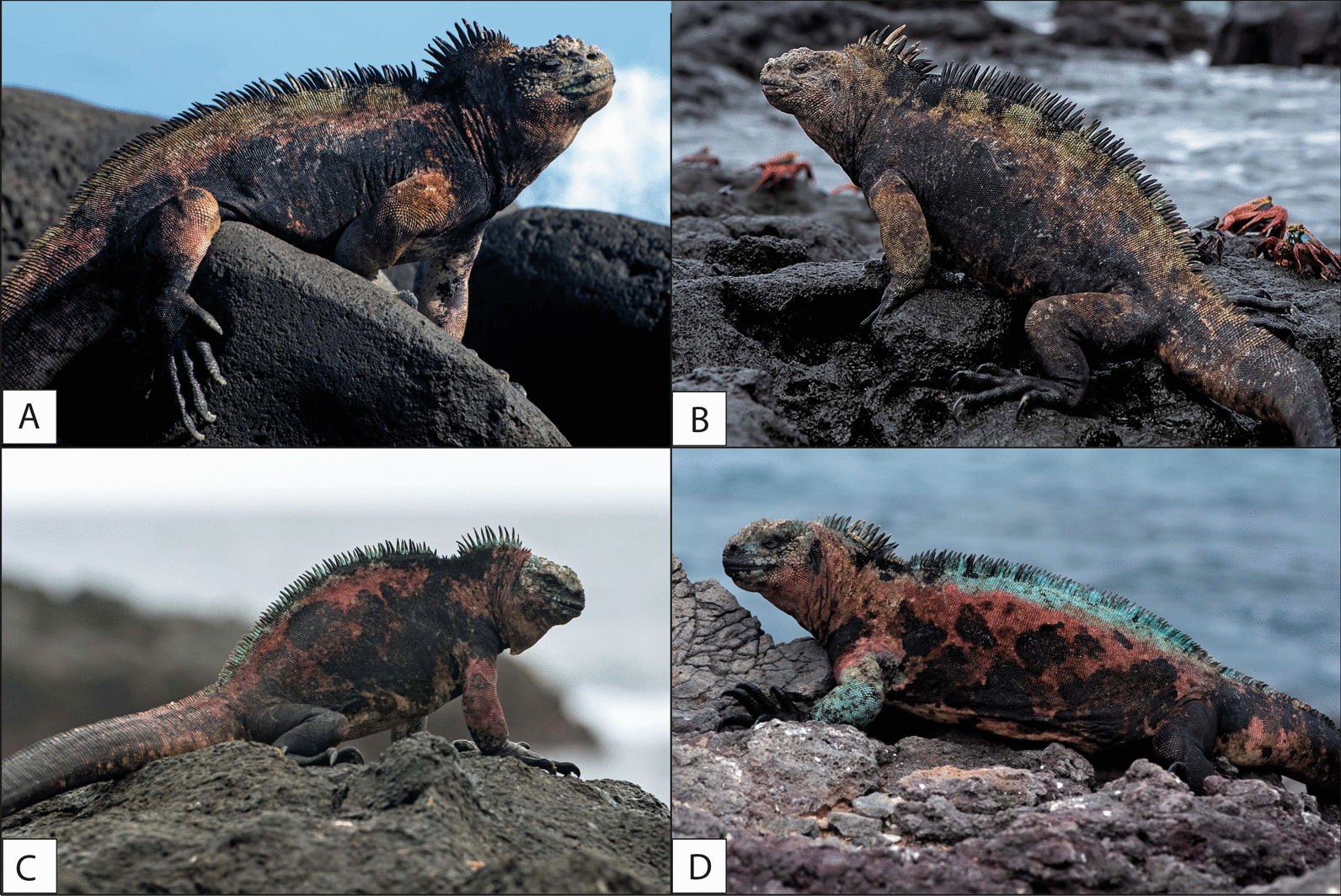
Images of the subspecies of marine iguana living at each of the sites surveyed. **A** *Amblyrhynchus cristatus mertensi* in La Lobería (mean SVL = 301.3 ± 92.6 mm), **B** *A. c. godzilla* in Playa Blanca (Punta Pitt) (mean SVL = 280.1 ± 58.3 mm), **C** *A. c. trillmichi* in El Miedo (mean SVL = 244.7 ± 23.6 mm), and **D** *A. c. venustissimus* in Gardner Islets (mean SVL = 263.5 ± 32.4 mm). Mean SVL taken from Miralles et al. 2017. Photographs: Andrés Mármol

We performed our surveys in the Galápagos Archipelago, a group of volcanic islands located ~ 1000 km from mainland Ecuador, South America. We chose four sites (colonies) distributed over three main islands to provide a good representation of conditions across the Galápagos; the islands studied were: San Cristóbal, Santa Fé, and Española (Fig. 1). La Lobería is situated on the south-western coast of San Cristóbal (0° 55′ 21.24″ S, 89° 37′ 4.55″ W), it comprises flat terrain (Fig. 4a) and harbors the subspecies *A. c. mertensi* (endangered; Fig. 5a). Generally, this area has calm sea conditions and is the only highly touristic place surveyed. Playa Blanca (also called Punta Pitt) is situated on the north-eastern coast of San Cristóbal (0° 41′ 42.04″ S, 89° 15′ 27.08″ W) and comprises flat rocky platforms (Fig. 4b); here *A. c. godzilla* occurs (critically endangered; Fig. 5b). This area usually has calm sea conditions but can become rough if northern swells are present. El Miedo, which harbors the *A. c. trillmichi* subspecies (critically endangered; Fig. 5c) is situated on the southeast coast of Santa Fé (0° 49′ 35.69″ S, 90° 1′ 43.68″ W) and has uneven terrain, with low cliffs (Fig. 4c). Here the sea is often rough. Gardner Islet is situated off the south-east coast of Española (1° 19′ 53.47″ S, 90° 17′ 56.69″ W), here *A. c. venustissimus* occurs (endangered; Fig. 5d). This location comprises irregular terrain of sharp rocks and some cliffs (Fig. 4d). Sea conditions at Gardner Islet vary daily, from calm to rough. Wind conditions in the archipelago can vary from mild to strong depending on the site and time of the year. We surveyed approximately 2 km along the coast in each location (2.0 km in La Lobería, 1.9 km in Playa Blanca, and 2.1 km in Gardner Islet), except for El Miedo, where we only covered 650 m since the abundance of the marine iguanas was relatively high and the colony compact.

The fieldwork took place during January 2021. This time of the year coincides with the mating season of the marine iguana (November–February) [53, 54]. This was chosen to improve sampling success, since at this time individuals congregate in mating groups (called leks) and tend to seldom leave these aggregations. Additionally, males tend to become brightly colored during these months and are easier to find and identify. Surveying took place between 08:00 to 14:00 h., corresponding to active hours described for marine iguanas [54].

### Aerial surveys and image collection

Images were collected using a DJI Mavic 2 Pro. This relatively cheap, consumer-level multi-rotor aircraft was chosen for its advanced camera (Hasselblad camera with 1-inch CMOS sensor with 20MP), good stabilization and ease of use; making it highly applicable for conservation purposes where it can be used by amateur pilots. This model was successfully used during a beach litter monitoring study [55] and for assessing water quality [56]. Our drone pilot was trained by an expert (GRT) for around 40 h. prior to undertaking the fieldwork; this proved sufficient for the safe and effective flight of the drones even from a moving boat. A co-pilot was also engaged to continuously watch the drone location, monitor for the presence of approaching birds, changing sea and weather conditions, as well as helping to launch and catch the drone. We preferentially launched the drones from a small fiberglass boat (7 m × 1.5 m in size). Only when conditions were very difficult (e.g., very rough seas or impassible rocky reefs), did we launch from land. Ideal weather conditions for the survey were good light levels (sunny or light clouds) and an absence of strong winds or rain; though we were often able to fly in non-ideal conditions. We flew the drones at a height of 20–25 m, a reasonable vertical distance so as not to disturb the fauna, and this altitude depended also on the body size of individuals monitored for a given population. For colonies in Santa Fé—where iguanas are on average 24 cm long (SVL) [57]— we flew at 20 m; on San Cristóbal or Española—where iguanas are on average bigger (30 cm SVL)—we flew at 25 m. To allow time to safely land the drone on the moving boat, we flew each battery, which represents one flight, on average between 21 and 24 min (from a maximum of 31 min.). The shorter flight times were a result of using an additional flotation device—to allow easy recovery in case of submersion—which added extra weight. We flew the drones at 2 m/s, covering an average distance of 300 m of coastline per flight; though this depended very much on the width of the area covered, as some iguana colonies extend much further inland than others. Typically, in this scenario, 1 km of coastline required between 1 and 1.5 h. (3–5 batteries) of survey time in the field when covered by a drone.

We collected high-resolution (5472 × 3648 pixels) still-images by performing automated and manual flights following a zigzag pathway (see Fig. 2) along the rocky coastline with the camera in a NADIR position (facing directly down). We used the app Map Pilot Pro (version 5.3.1) to create automatic flight paths ensuring the needed overlap between images for optimal reconstruction: forward overlap 80% and side-overlap of at least 60%; these parameters allow for accurate object identification (Agisoft Help Desk Software). Forward overlap is the percentage of overlap between one image and the next, and side overlap is between each leg of the flight. Map Pilot Pro generates flight paths and missions that can be saved and used to replicate surveyed areas exactly; this could be useful for future monitoring campaigns to allow comparisons between similar areas at different times. In parallel, we conducted manual flights replicating the automated flight mode using visual overlap calculation via the real-time drone imagery and map. We covered two sites using the app from land (La Lobería and El Miedo), and the other two were surveyed manually from the boat (Playa Blanca and Gardner Islet). We compared the outcomes of both methods after processing the images. The images were taken in JPEG/Exif format (av. 300 images per flight, total of 10,643 images collected) and the aperture and speed of the camera were set to automatic to enable constant rapid adjustment to changing light conditions. We also found it to be important to set up automatic focus or manually refocus the camera when taking every image to avoid blurred or unfocussed images.

### Image processing and counting

The aerial images include geographic coordinates acquired from the internal Global Positioning System (GPS) of the drone, therefore we used photogrammetry (a process that precisely defines the shape, size, and location of an object) to reconstruct our four surveyed sites in Agisoft Metashape Professional v.1.6 (http://www.agisoft.com). For this process, we align the geo-tagged images, build a dense point cloud, a mesh (depth map) and a texture to create an orthomosaic, which is a 2D geo-stitched reconstructed image (orthophotograph) or a georeferenced map. We set default parameters, except regarding output quality. Here we tested high-, medium-, and low-quality parameters, and compared the resolution obtained and time spent. Each of the four sites surveyed required 9–11 flights to complete, except for El Miedo which needed only three due to the compact area. This type of composite image avoids the analysis of each aerial image individually, therefore preventing double counting of individuals captured on multiple photographs. We then input the individual orthophotographs in the QGIS software v.3.12 (http://qgis.osgeo.org) carefully avoiding possible overlap between geographically consecutive orthophotographs. Here, we created a point-shapefile to identify, count, and geographically reference each marine iguana with a unique geomarker; these geomarkers also further ensure we did not count one individual twice in the whole orthophotograph. We drew a 4 × 4 grid over the orthophotograph to visualize a pattern for counting, which reduces the risk of missing individuals in the image. Since a high-resolution orthophotograph can be zoomed into enough to visualize the iguanas precisely, this decreases the risk of missing or overcounting individuals. Two skilled experts from our team examined the reconstructions and counted the iguanas for each site.

### Ground-based surveys

In parallel, we also counted marine iguanas *in-situ* by performing ground-based capture mark-resight (CMR) surveys in the four colonies. For CMR (two-occasion survey), iguanas were caught using a lasso on a pole and marked with an oil-based cosmetic paint, following the technique outlined in MacLeod et al. [29], resighting (second occasion of CMR) was undertaken after three or four days, with the drone-survey being performed in-between on the first and second occasion of the CMR. Due to the potentially disturbing effect of people within the colonies, ground-based counts were not performed at the same time as aerial surveys. However, weather conditions (sunny or lightly overcast) during aerial and ground-based surveys were similar, and both survey types occurred during the same time frame (between 08:00 and 14:00 h). The CMR method involves three counts: number of iguanas marked on the first occasion, number of marked iguanas resighted on the second occasion, and number of unmarked iguanas sighted on the second occasion. The second day of our CMR method is performed in precisely the same way as the “simple count” method used by the local conservation managers (GNP); we therefore took the total individual count (including both marked and unmarked animals) from the second CMR occasion as a proxy for the simple count method. We calculated the population-size estimate from the CMR data using Chapman’s modified Lincoln-Petersen index, in keeping with earlier work [29]. We chose these “traditional” methods as both have been previously used in this species, with CMR being a method that accounts for imperfect detection probability. When performed by the GNP, simple counts typically involve visually counting the number of animals present whilst walking through the colony. However, in high-density colonies, animals are often visually estimated rather than counted (pers. comm.). We then visually compared both ground-based methods to those from the drone-based approach by plotting the census estimates obtained against abundance in the colonies, since this is likely to influence the accuracy of the estimates. Additionally, we conducted an analysis of variance (ANOVA) among methods used to compare the time needed in the field and performed a post hoc Tukey HSD test to analyze the significance of these differences. Additionally, Chi-squared test was performed to compare the estimates gained using the traditional simple count method and the new drone-based method, the null hypothesis being that the counts were equal between the two approaches. Since the CMR method produces a range rather than a single number for the estimation of population size, we did not perform statistical comparisons against these outcomes.

## Data Availability

All data generated for this study are included in this article.
